# Determinants of Intention to Use Digital Technology for Older Adults by Environmental Dimensions

**DOI:** 10.1093/geroni/igab046.992

**Published:** 2021-12-17

**Authors:** Sangyoon Han, Seok In Nam

**Affiliations:** Yonsei University, Seoul, Seoul-t'ukpyolsi, Republic of Korea

## Abstract

Due to the COVID-19 pandemic, it is common to hear news of older adults being socially isolated due to difficulties in purchasing or accessing online services and in interacting with family or friends through video calling apps. Despite an increasing ease of access to digital devices, such access far from universal. Thus, digital inequality has become a serious problem for older adults. To understand why digital inequality issues are so relevant for older adults, we must understand older adults' entire life contexts and the potential of digital technologies in their lives. With these understandings, the purpose of this study was to explore the technology acceptance process and identify key precursors to acceptance of digital technology using the Technology Acceptance Model (TAM) 3 as a framework. This study used data from the 2018 Digital Divide Survey of the Ministry of Science and ICT. A total of 1,662 older adults (aged 55+) were analyzed using structural equation modeling with bootstrap sampling. Model fit indices (CFI = .928; SRMR = .074; RMSEA = .044) suggested an acceptable fit. Results indicated that two environmental dimensions, personal environment (self-efficacy and value recognition) and social environment (social norms and social support systems), had a significant impact on the intention to use technology both directly and indirectly. Furthermore, perceived usefulness and perceived ease of use mediated between environmental domains and the intention. This study indicates that providing appropriate digital support for older adults is important to achieve greater digital inclusion.

